# Sex-Specific Lifespan Extension and Anti-Obesogenic Effects of *Salicornia europaea* Extract Through Tor Signaling Modulation in *Drosophila*

**DOI:** 10.3390/nu17193065

**Published:** 2025-09-25

**Authors:** Navid Tahan Zadeh, Mirjam Knop, Lisa Marie Ulrich, Iris Bruchhaus, Roman Lang, Kai Lüersen, Gerald Rimbach, Thomas Roeder

**Affiliations:** 1 Department of Molecular Physiology, Kiel University, 24118 Kiel, Germany; navid.t.p.m@gmail.com (N.T.Z.); mknop@zoologie.uni-kiel.de (M.K.); lisamarieulrich@web.de (L.M.U.); 2 Developmental Glycobiology Section, NIDCR, National Institutes of Health, Bethesda, MD 20892, USA; 3 Bernhard-Nocht Institute for Tropical Medicine, 20359 Hamburg, Germany; bruchhaus@bnitm.de; 4 Leibniz Institute for Food Systems Biology, Technical University of Munich, 85354 Freising, Germany; r.lang.leibniz-lsb@tum.de; 5 Division of Food Sciences, Institute of Human Nutrition and Food Sciences, Kiel University, 24118 Kiel, Germany; luersen@foodsci.uni-kiel.de (K.L.); rimbach@foodsci.uni-kiel.de (G.R.); 6 DZL, German Center for Lung Research, ARCN, 24105 Kiel, Germany

**Keywords:** *Salicornia*, lifespan, *Drosophila*, Tor-signaling, extract, anti-obesogenic

## Abstract

**Background/Objectives**: Some marine plants and algae are known to exert health benefits. However, the long-term effects and underlying mechanisms of these health benefits are still poorly understood. For this reason, we have investigated an extract from the marsh samphire *Salicornia europaea* for its life-prolonging potential. **Methods**: We investigated the effect of an aqueous extract of *Salicornia europaea* (SEE) on the lifespan of several wild-type strains of *Drosophila*. In addition, we used deficient flies to elucidate the mechanism of the life-prolonging effects. Finally, we comprehensively phenotyped the treated animals. **Results**: Supplementing a standard diet with SEE extended the lifespan of different *Drosophila* laboratory strains by up to a third (37% in *w*^1118^ and 19% in *yw*). A total of 0.05% of SEE were ineffective, whereas 0.2% induced robust lifespan prolongation. This effect was strictly sex-specific, as the SEE application was completely ineffective in males, while prolonging life in females. We found that the body fat content of SEE-treated female flies was lower compared to controls. The extract also positively impacted the lifespan of flies fed a high-fat diet but not a high-sugar diet. SEE exhibited a lipase-inhibitory activity in vitro. Moreover, SEE counteracted aging-associated loss of intestinal barrier integrity. The sex-specific lifespan extensions induced by the SEE entirely depended on functional Tor signaling in the flies. Tissue-specific silencing of the Tor signaling pathway in different cellular compartments of the intestine reduced, but did not altogether abolish, the lifespan-prolonging effect in females. **Conclusions**: SEE is a promising candidate for a health-promoting intervention, as it induces lifespan-prolonging and anti-obesogenic effects in a sex-specific manner. These effects depend on functional Tor and partially on FoxO signaling. Future studies should identify the active compounds in the extract.

## 1. Introduction

Our diet significantly influences key aspects of our lives. This statement is especially true for the various facets of health. The diet’s composition, which can have positive or negative effects, is critical. Here, research often focuses on the relevance of macronutrient ratios [1,2,3]. In contrast, the health effects of other dietary components are far less well studied. Specific secondary metabolites have already been shown to impact human health positively. We can find these health-promoting nutritional components in various sources, including plants, algae, and fungi. Plants, algae, and fungi whose health-promoting properties are already known from traditional use are promising sources for in-depth analyses. Usually, only a few metabolites of these natural sources are responsible for the health-promoting effects. A typical example of such a pharmacologically active metabolite is rapamycin, a macrolide isolated from the fungus *Streptomyces hygroscopicus* [4]. Rapamycin has a health-promoting and life-prolonging bioreactivity by specifically intervening in the Tor signaling pathway and thus mimicking a calorie-reduced diet [5,6,7], a mechanism of action common to many lifespan-prolonging interventions.

Marine plants and algae are excellent candidates to expand the range of accessible nutritional sources that exhibit health- and life-prolonging properties. This assumption is based on the observation that healthy, or particularly long-living populations, have high proportions of certain marine plants and algae in their daily diet [8]. An example is the Okinawa region, where people consume a low-calorie diet with large amounts of plant-based marine products [9,10]. Following the hypothesis that specific diet components are responsible for the health benefits observed in these regions, we conducted a broad-based screening using a comprehensive plant and algal extract library [11]. We used the fruit fly *Drosophila melanogaster* as a screening model to quantify the effect of the different extracts on lifespan. *Drosophila* is ideally suited for such experimental strategies [12,13,14] because the flies have an organ composition very similar to ours, the metabolic characteristics exhibit a high degree of similarity to ours, and it is experimentally amenable to high-throughput screening of extracts and substances in terms of their effects on longevity [15]. Comprehensive screens like these have already allowed us to identify the life-prolonging effects of extracts of the marine algae *Saccorhiza polyschides* and *Eisenia bicyclis* and to elucidate their mode of action [16,17,18]. Apart from these two algae extracts, which have life-prolonging effects, only one other extract, an aqueous extract from the halophyte *Salicornia europaea* (SEE), showed this effect in our comprehensive screening experiment. *S. europaea*, also known as marsh samphire, sea asparagus, glasswort, or pickleweed, is a halophytic extremophile found primarily in intertidal salt marshes, mangroves, or beaches [19]. For centuries, it was used for food and medicinal applications. It exhibits antioxidant, anti-inflammatory, antidiabetic, and anticancer properties and can help slow aging [20,21]. It also has antibacterial activity [22].

Based on these initial results, we analyzed the potential health-promoting impact of aqueous *Salicornia europaea* extracts (SEE). We used Drosophila models to quantify the effects on lifespan and elucidated the underlying mechanisms. We showed that SEE extends the lifespan of flies by more than 30% in a sex-specific manner, only affecting females. Moreover, the extract reduced the triacylglyceride levels in flies and increased their survival on a high-fat diet, implying that the extract has lipid-lowering properties. Our data suggest that the aqueous extract of *S. europaea* contains bioactive compound(s) that target the Tor signaling pathway (Target of Rapamycin) to mediate the lifespan extension in female animals.

## 2. Materials and Methods

### 2.1. Plant Extraction

*Salicornia* extract was prepared according to the method described by Onur et al. [11]. In short, first fresh *Salicornia europaea* leaves were dehydrated overnight using lyophilization (Christ Alpha 2–4 LSC at −20 °C; Christ, Osterode, Germany), and the dried algal material was ground with an analytical mill (IKA Type A11 basic, IKA, Staufen, Germany). Three grams of ground alga were transferred into a test tube containing 30 mL of boiling, double-distilled water. After slight stirring for 5 min, the suspension was sonicated (1 min at step 3, Bandelin Sonoplus HD 2200; Bandelin, Berlin, Germany) and centrifuged for 2 min at 2000× *g* to remove non-solubilized material. The supernatant was filtered, and the soluble extract was stored frozen until use. A basal characterization of the extract has been performed in a recent project [17], where we identified known lead substances of aqueous SEE [21,23,24] to be present in high abundances, including the phenolic compounds caffeic acid and chlorogenic acid, the flavonoids kaempferol, corresponding glycosides and isorhamnetin, and betaine as well as saponin-derivatives [17].

### 2.2. Fly Husbandry

Wild-type adult flies were kept as previously described [17,25]. In brief, they were cultivated on a diet containing 5% (*w*/*v*) yeast extract, 8.6% (*w*/*v*) corn meal, 5% (*w*/*v*) sucrose, and 1% (*w*/*v*) agar-agar supplemented with 1% (*v*/*v*) propionic acid and 3% (*v*/*v*) nipagin. Adults, 3–5 days after hatching, were used in the experiments. All experiments were performed at 25 °C, a light-dark cycle of 12 h: 12 h, and 60% humidity. For most experiments, the wild-type strain *w*^1118^ was used. The following fly strains were used for the study: *w*^1118^ Bloomington stock ID #5905), Sir2-deficient (Bloomington stock ID #8838), Tor deficient (Bloomington stock ID #11218), *dfoxo*-deficient (foxo^21/21^, Forkhead-box O) (Marc Tatar lab), *y*^1^*w*^1118^ (Bloomington stock ID #111281), *P (Switch)106* (Ronald Kühnlein), *NP1-Gal4* (D. Ferrandon, Straßburg), *esg-Gal4*, *ppl-Gal4* (P. Leopold), *UAS-Tor.TED* (Bloomington stock ID #7013), *UAS-S6K-RNAi* (Bloomington stock ID #41702).

### 2.3. Lifespan Assay and Analysis

Lifespan assays were essentially performed as described earlier [26]. The mated female flies were separated into experimental groups and kept on the food described above. Vials were regularly changed every 2 days, and dead flies were counted daily. For the high-sugar diet, the sucrose concentration was increased to 30%, and for the high-fat diet; the concentrated medium was supplemented with 20% coconut oil. To supplement the food with the algal extract, the aqueous extract was added on top of the food in the experimental vials at the final concentration of interest. Experiments with food dye showed the formation of a concentration gradient with the highest concentrations at the surface of the food. Then, the water is allowed to evaporate to dry the food. At least 100 animals were used per experiment, with 10 animals per vial and more than 10 vials for each experiment.

### 2.4. Body Fat Quantification

The whole-body triacylglycerol (TAG) content was measured using a coupled colorimetric assay as previously described [26,27]. In short, samples were collected (5 females per sample) and weighed. 1 mL PBS/Tween-20 (0.05%) was added to the samples, and they were homogenized in a bead ruptor apparatus (BioLab Products, Bebensee, Germany) for 2 min at 3.25 m/s. Next, samples were centrifuged for 3 min at 3000× *g*, and the supernatant was transferred to new tubes. The supernatant was heat-inactivated at 70 °C for 10 min and centrifuged for 3 min at 2500× *g*. A total of 50 μL of each sample was added to a 96-well plate, and the absorbance was measured at 500 nm (T_0_). 200 μL prewarmed triglyceride reagent was added to each well and incubated for 30 min at 37 °C with mild shaking. The absorbance was again measured at 500 nm (T_1_). The TAG concentration was determined by subtracting T_0_ from T_1_. The TAG content was quantified using a triolein-equivalent standard curve.

### 2.5. Glucose Measurement and Protein Content Analysis

The body glucose levels were determined using a Glucose (HK) Assay Kit (GAHK-20, Sigma-Aldrich, Taufkirchen, Germany) according to the manufacturer’s instructions with minor modifications. Samples were collected (5 female flies per sample), weighted, and homogenized using a bead ruptor apparatus (BioLab Products, Bebensee, Germany) for 2 min at 3.25 m/s. For the glucose measurement, the supernatants were heated for 10 min at 70 °C and then centrifuged for 3 min at 4 °C. A total of 30 μL of the supernatant was added to a well of a 96-well plate. 100 μL of the HK reagent was added to each well and the plate was incubated at room temperature for 15 min. Then, the absorbance was measured at 340 nm. The glucose content was calculated using a glucose standard curve. The samples were centrifuged for 1 min at 1000× *g* at 4 °C to determine the protein content. The supernatant was transferred to a new 1.5 mL tube and centrifuged at 6000× *g*, at 4 °C for 10 min. The supernatant was again transferred to new tubes and centrifuged at maximum speed, at 4 °C for 10 min. The Pierce BCA Protein Assay Kit measured the protein content according to the manufacturer’s instructions.

### 2.6. Starvation, Desiccation, Paraquat Resistance, and Fecundity Assays

To assess flies’ resistance to starvation, flies were transferred to vials containing 1% agar after feeding on food of interest. The survivorship of the flies, which were kept at 25 °C, 60% humidity, and a 12/12 h light/dark cycle, was recorded every two hours. To determine the resistance to desiccation, flies were transferred to empty vials after being fed the food of interest. Again, the flies were kept at 25 °C and 60% humidity and a 12/12 h light/dark cycle. The survival of flies was monitored every 1 to 2 h.

For the paraquat assay, flies were treated with the extract for 3 weeks and then transferred to food supplemented with 20 mM paraquat (methylviologen) without extract addition. The number of dead flies was counted for 2 weeks.

To determine egg production, mated female flies were kept in vials containing the food of interest and the laid eggs were counted daily for 14 days.

### 2.7. Food Intake

The food consumption of flies was measured using the consumption-excretion method [28]. The fly medium or blue dyed medium (0.5% (*w*/*v*) Brilliant Blue FCF food dye; E133) was dispensed into caps of 2 mL screw cap vials. For adaptation, individual flies were transferred to 2 mL screw cap vials with CM. After a few hours of feeding on the concentrated medium, the flies were transferred to 2 mL vials with blue-dyed food. After 24 h of feeding on blue-dyed food, 3 ceramic beads and 500 μL H_2_O were added to the vials. The samples were homogenized using a bead ruptor (OMNI International, OMNI Bead Ruptor 24, Kennesaw, GA, USA) for 90 s at 3.25 m/s. The homogenized samples were centrifuged at 3000× *g* for 3 min to deposit the tissue debris. Then 200 µL of the supernatant was added to clear 96-well plates, and the absorbance was measured at 630 nm.

### 2.8. Smurf Assay

Animals were reared on fly medium for 20 and 27 days before they were transferred to a dyed medium containing 1.5% (*w*/*v*) Brilliant Blue FCF in CM. After 24 h, the animals were inspected and counted as Smurf when the blue dye was observed outside the intestine [29].

### 2.9. Statistics

Statistical analyses and plotting figures were performed using GraphPad Prism versions 7 and 8. For lifespan analyses, a Log-rank (Mantel–Cox) test was used. For other experiments, the data were first tested for a normal Gaussian distribution using the Shapiro–Wilk normality test. Subsequently, an unpaired *t*-test was used for data exhibiting a normal distribution, and the Mann–Whitney test was used for the other data.

## 3. Results

### 3.1. Salicornia Extracts Extend the Lifespan of Drosophila melanogaster in a Sex-Dependent Manner

**Figure 1:**
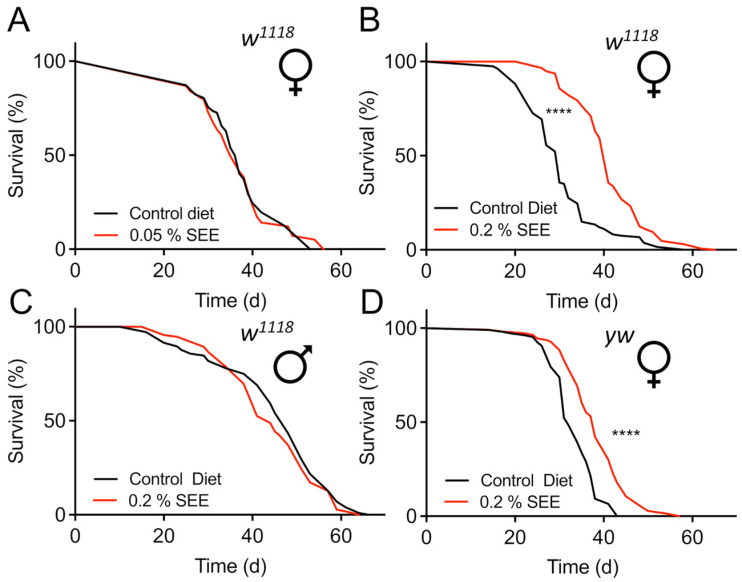
Effect of SEE on lifespan in flies of different sexes. (**A**) Effect of supplementation with 0.05% of SEE on the lifespan of *w*^1118^ female flies compared with untreated animals (N = 100, the median lifespan of SEE-treated flies = 35 d, the median lifespan of untreated flies = 37 d). (**B**) 0.2% of SEE effects on lifespan of female *w*^1118^ flies (N = 150, median lifespan of SEE-treated animals = 41 d, the median lifespan of the control group = 30 d). (**C**) Comparison of the lifespans of male *w*^1118^ flies subjected to 0.2% SEE and those receiving a non-supplemented diet (N = 100, median lifespan of both groups = 41 d). (**D**) SEE increased the lifespan of *yw* female flies (N = 100, median lifespan of flies treated with SEE = 38 d, median lifespan of control = 32 d). SEE: *Salicornia europaea* extract, ns: not significant, **** *p* < 0.0001.

We identified an aqueous extract (Supplementary Table S1) of *S. europaea* (SEE) during a larger screen of plant and algal extracts for their life-extending potential using fruit flies as a model system. To further characterize and verify this lifespan-prolonging effect, we used two different concentrations (0.2% and 0.05%) of this extract and measured lifespan in cohorts of mated females of the *D. melanogaster w*^1118^ strain. Animals subjected to 0.05% SEE did not show any lifespan prolongation (Figure 1A). However, application of the extract at the higher concentration of 0.2% significantly increased median lifespan by 36.6% (Figure 1B, *p* < 0.0001). We also tested cohorts of mated males, but we did not observe any lifespan extension by supplementation with 0.2% SEE in males (Figure 1C). To exclude the possibility that the observed effects on lifespan are only strain-specific, we tested a second *Drosophila* strain, namely *yw*. Although both strains are commonly used laboratory strains that function as background for most genetically modified *Drosophila* strains, they differ substantially in, e.g., their behavior [30]. Here, we could also show a robust lifespan prolongation by 16.1% induced by 0.2% SEE (Figure 1D, *p* < 0.0001, Table 1). The increases in lifespan were not only seen for the median lifespan but also the maximal lifespans (i.e., the 10% longest-living animals) in both *w*^1118^ (49 d and 53 d; *p* < 0.0001) and *yw* animals (43 d and 49 d, *p* < 0.0001).

### 3.2. Salicornia-Treated Fruit Flies Are Lighter and Have Reduced Triacylglyceride Levels

**Figure 2:**
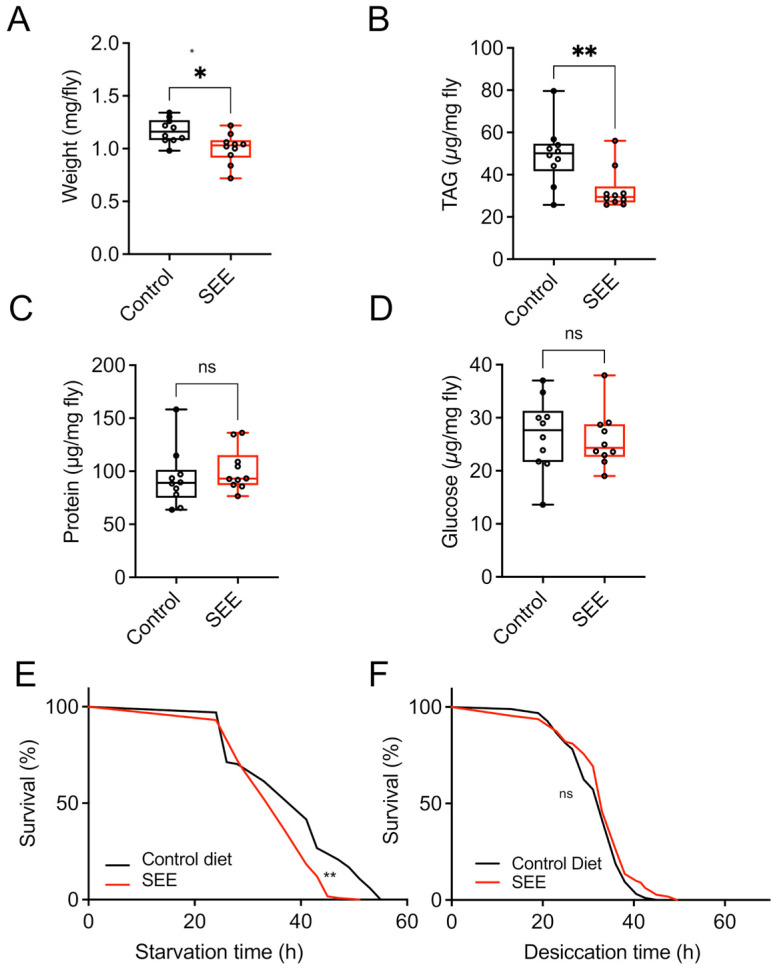
Body composition and resistance towards starvation and desiccation of female flies in response to 0.2% SEE. (**A**) Body weight of female flies treated with SEE or not (N = 10, box plot showing median and minimum as well as maximal values). (**B**) Effects of SEE treatment on body fat (triacylglycerol) levels (N = 10). (**C**) Quantitative analysis of the protein content of SEE-treated females and their matching controls (N = 10). (**D**) SEE effects on the glucose content of flies (n = 10). (**E**) Starvation resistance of flies treated for three weeks with SEE. (N > 100, median survival of SEE-treated flies = 37 h, median survival of untreated flies = 41 h). (**F**) Desiccation resistance of SEE-treated female flies (N = 100, median survival of SEE-treated flies = 33 h, median survival of untreated flies = 33). SEE: *Salicornia europaea* extract, ns: not significant, * *p* < 0.05, ** *p* < 0.01.

To assess whether feeding 0.2% SEE affects the body composition of mated female flies, we quantified parameters such as body weight, triacylglyceride levels (TAG), protein, and glucose content after three weeks of treatment. Regarding body weight, the SEE-treated group was slightly lighter than the non-treated animals (Figure 2A, *p* = 0.01). We also quantified the TAG levels and found substantially lower TAG amounts (Figure 2B, *p* = 0.007), while for the body protein content, the experimental groups did not show any significant differences (Figure 2C). Moreover, we found no significant changes in glucose levels in SEE-treated animals (Figure 2D). Finally, because SEE-treated flies had lower TAG levels, we analyzed the starvation resistance, which is usually directly associated with the body fat content [27]. In good agreement, SEE-treated animals showed a reduced starvation resistance with a median survival of 37 h compared to controls with a median survival of 41 h (*p* < 0.001; Figure 2E). We next analyzed if the addition of the SEE changed the survival in response to desiccation stress with a similar median lifespan for the control group and flies after two weeks of treatment (Figure 2F, *p* = 0.0633).

### 3.3. Effect of SEE Extract on Energy Metabolism

**Figure 3:**
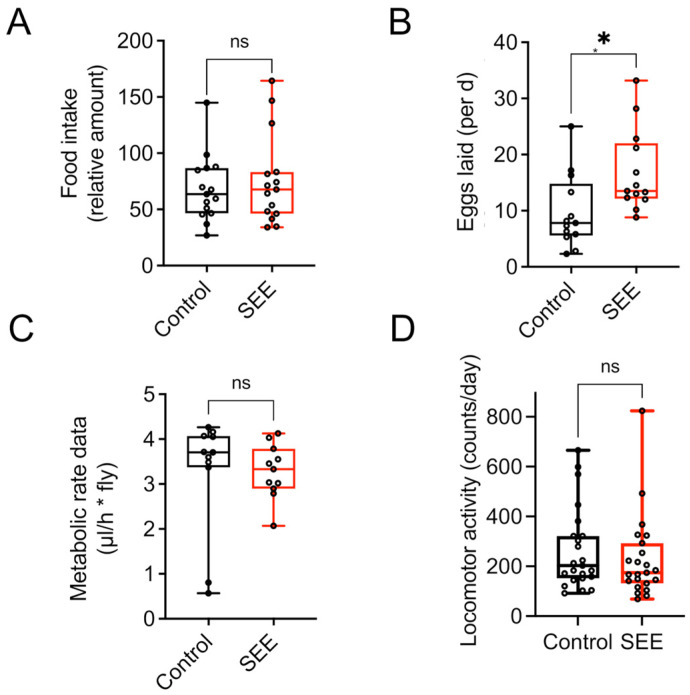
Energy intake and energy expenditure in response to 0.2% SEE. (**A**) Food intake of female flies in response to SEE treatment during a 24 h period (N = 15). (**B**) Influence of SEE on egg production over a period of 14 days, shown as eggs laid per day (N = 10). (**C**) Metabolic rate of flies subjected to SEE (N = 10). (**D**) The locomotor activity of adult flies either subjected to SEE treatment or not was quantified using a DAM monitor during 24 h periods (N = 25). ns: not significant, * *p* < 0.05.

We quantified the nutritional intake using the blue food assay to exclude indirect effects caused by reduced uptake, thereby leading to an induced caloric restriction with its lifespan-prolonging effects. There were no statistically significant differences in food consumption for 24 h between the experimental groups (Figure 3A, *p* = 0.5681), which implies no change in energy intake. We also quantified the most energy-consuming trait in *Drosophila*, egg production, to evaluate if the observed lifespan extension compromised other health measures or came at the cost of reduced fecundity. The measurements revealed that the SEE-treated flies showed a higher reproductive output as evidenced by higher numbers of eggs laid in the two-week monitoring period (Figure 3B, *p* = 0.0146). In addition, we analyzed the animals’ metabolic rate and found that the experimental groups showed no significant alteration in CO_2_ production (Figure 3C). Finally, we quantified physical activity as a measure of energy expenditure over 24 h periods using the *Drosophila* activity monitor. Here, no significant differences were seen between the experimental groups (Figure 3D).

### 3.4. Stress Resistance to High-Fat Dieting Is Enhanced After SEE Treatment

**Figure 4:**
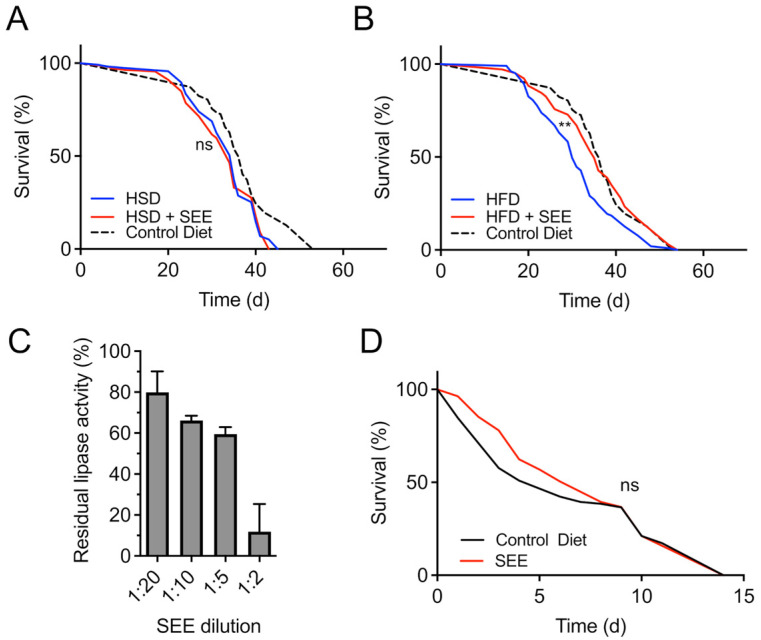
Stress resistance to high-fat dieting is enhanced after 0.2% SEE treatment. (**A**) Impact of SEE on the survival of flies subjected to a high-sugar diet (N > 110, the median lifespan of SEE-treated = 34 d, the median lifespan of untreated flies = 35 d). (**B**) The survival of flies fed with SEE and subjected to a high-fat diet (N = 100, the median survival of SEE-treated flies under HFD = 35 d, the median survival of untreated flies under HFD = 30 d). (**C**) SEE extract affects lipase activity in a dose-dependent manner (N = 3). Listed are different SEE dilutions and their effect on lipase activity. The bar plots show mean values ± S.D. (**D**) The median survival of flies under paraquat stress is not affected by pre-feeding a SEE-containing diet for 3 weeks (N > 100, the median survival for SEE-treated flies = 7 d, the median survival of control flies = 6 d. SEE: *Salicornia europaea* extract, ns: not significant, ** *p* < 0.01.

Next, we tested the interaction of SEE with two major nutritional stressors, namely high-fat and high-sugar diets. Flies treated with 0.2% SEE had the same median lifespan as the control group if confronted with a high-sugar diet (Figure 4A, *p* = 0.7368). In contrast, when rearing flies on food containing 20% coconut oil (high-fat diet), we found that the SEE addition prolonged the median lifespan by about 16.6% from 30 d under control conditions to 35 d in response to SEE application (Figure 4B; *p* < 0.0014). The maximum lifespan increased significantly from 48 d in controls to 51.5 d in the SEE group (*p* = 0.0180). Interestingly, these results align with the extracts’ robust dose-dependent lipase inhibiting activity indicating a mode of action for the extract, which involves targeting the intestine lipases thus reducing intestinal absorption of dietary fat. When we employed an in vitro assay to examine the lipase-inhibiting properties of the SEE, a 1:2 dilution of SEE reduced lipase activity by almost 90%, and even a 1:20 dilution led to an enzyme inhibition of almost 20% (Figure 4C).

We also examined the effect of the extract upon paraquat treatment as an oxidative stress inducer. In this condition, we did not observe a lifespan-extending impact of the extract (Figure 4D).

### 3.5. Mode of Action of the Lifespan Prolonging Effects of Salicornia Extract

**Figure 5:**
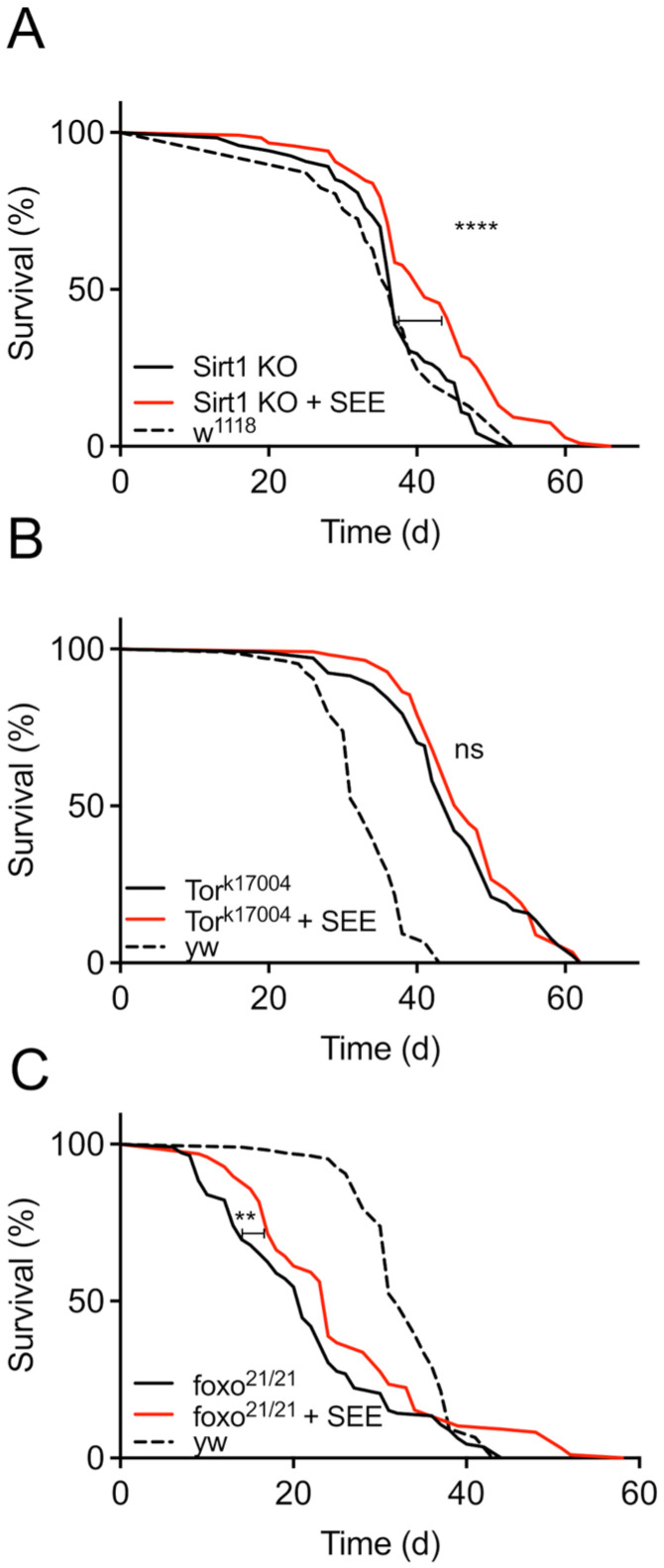
Signaling pathways potentially involved in mediating lifespan extension by SEE. (**A**) Lifespan of *Sirt1*-deficient flies in response to 0.2% SEE in the diet compared with the untreated animals as well as with the genetic background controls (*w*^1118^) (N = 110, median lifespan of SEE-treated *Sirt1*-deficient flies = 41 d, median lifespan of *Sirt1*-deficient flies = 37 d). (**B**) Lifespan effects of SEE in *Tor*-deficient flies compared to non-treated flies as well as to the genetic background (*yw*) (N = 100, median lifespan of *Tor^K17004^* flies = 45 d, median lifespan of *Tor^K17004^* flies fed with SEE = 46 d). (**C**) SEE effects on lifespan of *dfoxo*-deficient animals compared with non-treated ones and with the genetic control (*yw*) (N = 100, median lifespan of SEE-treated foxo-deficient flies = 24 d, median lifespan of *dfoxo*-deficient flies = 21 d). ns: not significant, ** *p* < 0.01, **** *p* < 0.0001.

To elucidate potential mechanisms by which SEE extends the lifespan of female flies, we next focused on the major signaling pathways that have repeatedly been shown to determine significant aspects of the aging process. These are the Sirtuin-, the Insulin/IGF signaling (IIS)-, and the Tor/TOR signaling pathways [31,32]. Since activation of the Sir2 pathway has life-prolonging properties in *Drosophila* [31], we employed *Sir2*-deficient flies and observed an almost identical lifespan on the control diet compared with the genetic background (*w^1118^*). Moreover, SEE treatment prolonged the lifespan of the *Sir2*-mutants, indicating that *Sir2* is not necessary to transmit the effects of SEE on lifespan (Figure 5A). Furthermore, we analyzed the Tor/TOR signaling pathway by targeting the central molecule of this pathway using the hypomorphic allele *TOR^k17004^*. As expected [33], we observed an increased lifespan in flies with a *TOR^k17004^* background compared to the matching control (*yw*). However, SEE-treatment did not further positively affect lifespan in these flies, showing that Tor-signaling is necessary for mediating the lifespan prolongation in response to SEE (Figure 5B). Since the Tor signaling and IIS are interwoven [17], we also assessed the effect of SEE on the latter signaling pathway. To this end, we evaluated the role of the FoxO transcription factor, which is not only a proxy of IIS, but also a protein directly involved in lifespan determination. *dFoxo*-deficient flies showed a significantly reduced lifespan compared to their matching controls (Figure 5C). Administration of SEE extended the lifespan of these animals, which indicates that FoxO signaling is not required to mediate the SEE effects on lifespan (Figure 5C). This increase in lifespan is substantially smaller than in wildtype flies, implying that FoxO signaling might be involved to some extent in SEE-mediated lifespan prolongation.

**Figure 6:**
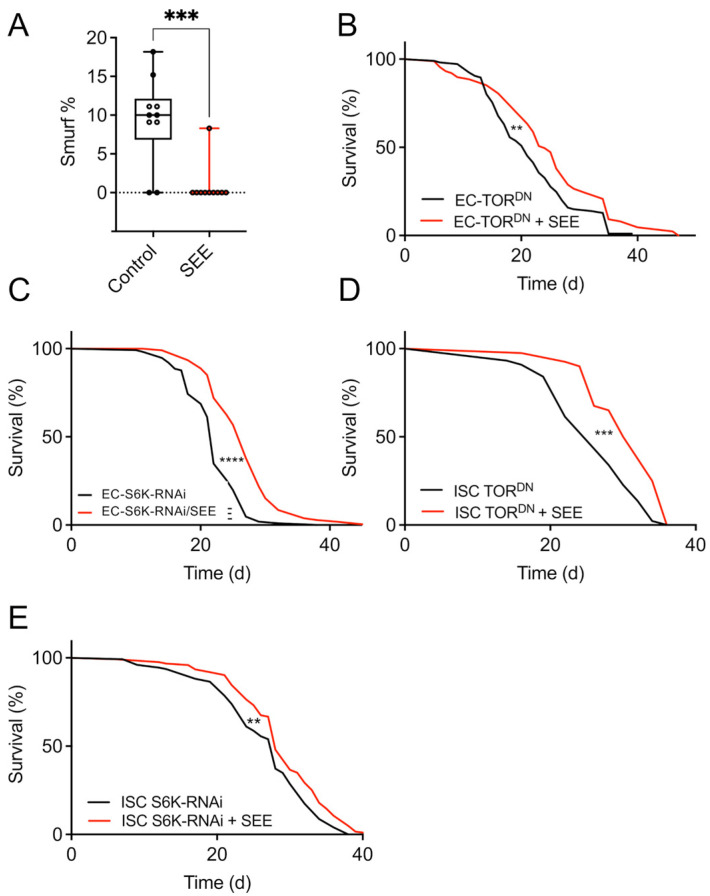
Tor/S6K signaling in different cell types of the intestine impacts SEE-induced lifespan effects. (**A**) Smurf analysis of flies fed a normal diet, or a diet supplemented with 0.2% SEE for 20 d. Data are shown as boxplots with whiskers indicating minimal and maximal values (N = 10 with 20 animals per biological replicate). (**B**) The impact of SEE supplementation on the lifespan was examined in female fruit flies whose Tor signaling was reduced only in enterocytes (*Np1-Gal4/tuBGal80>UAS-Tor.TED*). Flies were maintained under control conditions or treated with 0.2% SEE (N = 83). (**C**) The lifespans of fruit flies with reduced S6K expression in their enterocytes (*Np1-Gal4/tuBGal80>UAS-S6K-RNAi*) were determined in the presence and absence of 0.2% SEE (N = 101). (**D**) The SEE effect on lifespan was tested in flies with dominant negative Tor expression (*Tor^DN^*) in ISCs and EBs (*esgGal4, UAS-GFP; tub-Gal8>UAS-Tor.TED*) (N = 45, median lifespan of *esgGal4, UAS-GFP; tub-Gal80>UAS-Tor.TED* + SEE = 31 d, *esgGal4, UAS-GFP; tub-Gal80>UAS-Tor.TED* = 26 d) and (**E**) in flies with RNAi-depleted S6K in ISCs and EBs (N > 120, the median lifespan of *esgGal4*, *UAS-GFP*; *tub-Gal80>UAS-S6K-RNAi* + SEE = 28, the median lifespan of *esgGal4*, *UAS-GFP*; *tub-Gal80>UAS-S6K-RNAi* = 28). ** *p* < 0.01, *** *p* < 0.001, **** *p* < 0.0001.

To further narrow down the mode, especially the side of action, we focused on the intestine and the fat body as the most critical organs in mediating lifespan extension by nutritional interventions [17,34]. To test if the intestine is relevant for the lifespan extension mediated by SEE application, we performed a Smurf assay, which is a direct proxy of intestinal health and correlates nicely with lifespan [29]. Here, at 20 days, 9.5% of the control group were smurf-positive, while the corresponding number for the SEE-treated group was only 1% (Figure 6A). This difference was statistically significant (*p* < 0.005). We then manipulated Tor/TOR signaling in the enterocytes (*Np1-Gal4>UAS-Tor^DN^*) by induction of a dominant negative *Tor* allele (*Tor^DN^*). Here, SEE supplementation led to an extended lifespan of female flies (Figure 6B). A similar result, i.e., a SEE-mediated lifespan extension, was obtained when we tested flies, in which Tor signaling was diminished through RNAi-mediated depletion of downstream S6K in enterocytes (*Np1-Gal4>UAS-S6K-RNAi)* (Figure 6C). Furthermore, we used a driver specifically addressing intestinal stem cells and enteroblasts (*esg-Gal4*). Attenuation of Tor signaling using the Tor dominant negative allele in these cells did not abolish the beneficial effects of the extract (Figure 6D). However, the lifespan prolongation was relatively small. Similarly, silencing of S6K in these cells (*esg-Gal4>UAS-S6K RNAi*) did not rescue the lifespan extension phenotype (Figure 6E).

**Figure 7:**
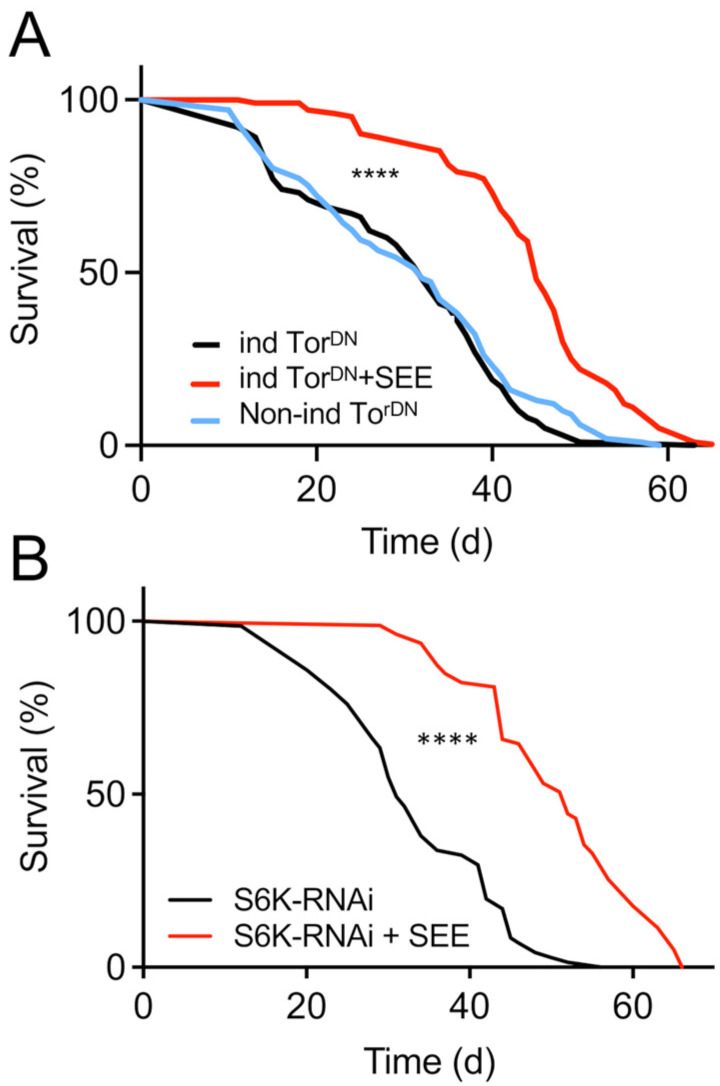
Fat body Tor/S6K signaling has no impact on lifespan prolongation induced by SEE. (**A**) Lifespan of flies with abolished Tor signaling (*Tor^DN^*; ind for induced) in the fat body (*P(GS)106>UAS-Tor.TED*) with and without SEE treatment, and of non-induced flies of the same genotype (N = 100, median lifespan of uninduced flies = 32 d, median lifespan of induced flies = 32 d). (**B**) SEE effects on lifespan of flies with reduced S6K signaling in the fat body compared with non-treated animals (*ppl-Gal4>UAS-S6K-RNAi*) (N = 75, median lifespan of *ppl-Gal4>UAS-S6K-RNAi* = 31 d, median lifespan of *ppl-Gal4>UAS-S6K-RNAi* + SEE = 52 d). **** *p* < 0.001.

To evaluate if Tor-signaling in the fat body is required for the SEE-induced lifespan extension, we used the RU486-inducible abdominal fat body-specific driver (*P (106) GS*) for expression of the *Tor^DN^* allele (Figure 7A). SEE application led to a significant increase in lifespan under these conditions, implying that Tor signaling in the fat body is not required for the SEE effect on lifespan (Figure 7B). We also silenced S6K by RNAi targeted to the fat body of female flies. In good alignment, these animals showed prominent lifespan extension by SEE application (Figure 7B), suggesting the mode of action of the extract was uncoupled from downregulation of S6K in the fat body.

## 4. Discussion

We found that an aqueous extract of the marsh samphire (*S. europaea*) (SEE) significantly prolongs the lifespan of female *D. melanogaster*. This significant lifespan extension by 30–40% is seen for mean and maximum lifespan. Since we observed this effect in more than one *Drosophila* strain, the extract possibly exhibits similar positive effects on fruit flies in general. Although the extract exhibited a positive effect on different strains, the life-extending effect was less pronounced in *yw* compared to *w*^1118^, which might reflect different metabolic properties in these different strains. Only females show this SEE-mediated effect on lifespan, whereas male flies did not benefit from a SEE treatment. Comparable sex differences in response to a life-prolonging intervention have already been shown in other studies [35,36]. Here, mice and *Drosophila* were the main models. Still, a general picture emerged that females benefit more from lifespan-extending interventions than males, regardless of whether they are pharmacological or nutritional [35,36,37], which is in line with higher stored fat amounts and higher food intake in females [38]. Especially the increase in food intake might be operative in mediating these sex-specific effects, as more compound is ingested. We found the extract modulates lipid metabolism by reducing the whole-body TAG levels. These results might help to explain the female-specific effects since lipid metabolism is differentially regulated in male and female flies as the males [39,40]. In a recent study focusing on the brown alga *E. bicyclis* extract, we already showed a sex-specific lifespan extension in response to this nutritional/pharmacological intervention [17]. This clear dependence differs, for example, from a recent study with extracts from a brown alga *S. polyschides*, in which both sexes showed similar degrees of lifespan extension [16]. Despite this wealth of studies showing sex-specific effects on longevity, the underlying mechanism for these differences usually remained unclear. However, one of the few mechanistic studies attributed the differential impact of a life-prolonging pharmacological intervention to specific properties of the *Drosophila* intestine [34]. A similar mechanism is also operative in response to the SEE extract, as our data implies that the lifespan-prolonging effects depend on modifications within the gut. As already pointed out, male flies lack the Tra protein which is a key regulator of fat storage by influencing the whole-body triglyceride levels. Thus, this discrepancy in lipid metabolism can be one possible explanation for the female-specific effects of the extract [39,40].

We also elucidated the underlying molecular mechanism responsible for SEE-mediated lifespan extension. In the process, it turned out that the Tor/TOR signaling pathway is indispensable for these very effects. This finding is in line with several studies in which the importance of the Tor/TOR signaling pathway for all age-associated processes has been worked out [41,42,43]. Consequently, targeting this signaling pathway to enhance life- and health-span is a promising strategy. In further experiments, we tried to identify the organ responsible for the lifespan extension induced by SEE. The intestine and the fat body were the prime candidates for this role. Our smurf experiments showed that SEE treatment positively affects intestinal functionality. This reaction is relevant as barrier loss in the intestine is a hallmark of aging and is closely associated with premature death [29,44]. Silencing Tor/TOR signaling in different gut compartments showed that the SEE-induced response was not completely abolished. Still, it was substantially reduced in animals experiencing blockade of Tor/TOR signaling in intestinal stem cells. This dependency implies that the intestine is involved in SEE-induced lifespan prolongation but is not the only target organ. Coming back to the different cell populations of the intestine, we found that the contribution of the intestinal stem cells is more important than that of the enterocytes. This result implies that modifying the biology of intestinal stem cells is more important, which is in line with results showing the central role of this cell population for organismal aging [45]. The fat body has often been shown to be relevant for lifespan-extending interventions [31]. Nevertheless, it is dispensable for mediating the SEE-induced effects on lifespan. Moreover, we found a lipase-inhibiting activity of SEE, presumably not the primary reason for the lifespan prolongation under control conditions, but might have an additional beneficial effect, especially in high-fat dieting situations.

If we compare the main results of the current study using a *Salicornia* extract with those of a previous one employing an *E. bicyclis* extract [17], several commonalities are striking (Table 2). The effects observed in both sets of experiments comprise strict sex-specificity and dependence on Tor signaling. The substantially reduced life-prolonging properties in a *dfoxo*-deficient background imply that FoxO signaling is necessary but not essential for mediating the SEE effects. This differs from the *Eisenia* extract, where the lifespan prolongation is strictly FoxO-dependent. Furthermore, the different extracts induce increased resistance to different nutritive stress situations. While the *Eisenia* extract protected particularly well against a high-sugar diet and hardly affected a high-fat diet, the *Salicornia* extract was particularly effective in mediating positive effects when the animals were confronted with a high-fat diet. This result implies that the molecular mechanisms underlying the effects of *E. bicyclis* and *S. europae* extracts share the requirement of active Tor/TOR signaling but differ in how they interfere with this signaling pathway.

*Salicornia* species are potential food sources and a source for pharmaceutically active compounds [19]. An essential reason for using *Salicornia* in this way is the almost worldwide availability of this plant. Salicornia species have been studied intensively as a source for pharmaceutically relevant phytochemicals. The abundance of active compounds in the extract might account for the unexpected increased fecundity of SEE-treated female flies despite reduced fat content and increased lifespan, reminiscent of the pro-longevity and -fecundity effects of the TCA intermediary metabolite citrate as the flies fed with 1% supplementary citrate had increased activity and fecundity [46]. In line with this assumption, SEE is enriched with two TCA metabolites: isocitrate and fumaric acid [46]. Although this is a tempting hypothesis, it must be pointed out again that it is still just that: a hypothesis that needs to be verified in future.

The metabolic effects of *Salicornia* products are highly relevant for this work, with particular emphasis on antidiabetic properties and those leading to a reduction in fat storage [47]. Lowering fat deposition was associated with interference with SREBP1-mediated processes induced by different components found in the *Salicornia* extract. This reduction in fat storage is entirely in line with our observations, showing a potent inhibition of lipase activity. Moreover, immunomodulatory and anti-inflammatory effects were identified, predominantly mediated by polysaccharides of *Salicornia* extracts [48]. A similar effect as a hypolipidemic agent that can potentially reduce hepatic accumulation was reported recently using *Salicornia* extracts [49]. Nutritional interventions that prolong lifespan without compromising fertility are rare to find [50]. In the current example of the SEE, a resource allocation shift induced by the SEE towards egg production at the expense of store lipids could be an explanation.

The anti-obesogenic activity of the SEE is a highly desirable feature. The search for plant extracts and other sources of compounds with precisely these properties has high priority [51]. Here, different mechanisms are operative [52]. Lipase inhibition, as observed in the SEE, appears to be a significant mechanism of anti-obesogenic effects induced by natural products [53]. Despite this compelling bioactivity of the SEE as an anti-obesogenic source, the lifespan-prolonging effects are operative through a different mechanism, which is Tor-dependent. The control diet, where SEE induced a substantial lifespan prolongation, is not rich in lipids, further pointing to different mechanisms underlying the lifespan prolongation and the anti-obesogenic effects.

The composition of *Salicornia europaea* extracts has been extensively described in a series of recent publications [21,54,55,56]. As we have already conducted a comparative analysis of Salicornia extract composition about *Eisenia bicyclis* and *Sacchoriza polyschides* extracts [17], we have decided not to analyze the extract components further for this study. Based on metabolome analyses published by us and others, we will focus below on metabolites that we detected in higher abundances in the SEEs. As the three lifespan-prolonging extracts exert their activities through different mechanisms, we will discuss especially those metabolites that we found explicitly in the *Salicornia* extracts and are known to affect lifespan. Chlorogenic acid, tuberonic acid, melleolide, isorhamnetin, or kaempferol are especially relevant here. Chlorogenic acid, for example, is one of these substances that showed an apparent life-prolonging effect in *C. elegans*. Here, interaction with insulin signaling and the Akt-FoxO axis was required to unfold these effects [57,58]. In addition, isorhamnetin, a methylation product of quercetin, also enhances lifespan in *C. elegans* and is resistant to stressors [59]. A Lotus (*Nelumbo nucifera*) stamen extract containing several of these compounds, found explicitly in the *Salicornia* extract, showed a robust delay of aging in yeast. Here, kaempferol and isorhamnetin must be mentioned [60,61]. Thus, some of the lifespan-prolonging effects of the *Salicornia* extract could be mediated through the compounds discussed above that are present at high concentrations in the SEE. We intend to use this information, which ultimately paves the way for identifying the lifespan-mediating metabolites, for this very purpose in follow-up studies applying purified test compounds alone and in combination. This result is beyond the scope of this manuscript and should be the subject of future studies. Finally, future studies should not only clarify potential lifespan extension effects in mammals or even humans but also assess possible toxicological concerns of the extract and its components.

As with all experimental studies, this study has its inherent limitations. This is particularly true of animal studies, which typically consider only a limited range of genetic diversity. To counter this potential bias, we used several different laboratory strains. This enables us to make preliminary generalizations about the SEE effects on *Drosophila*. As we have not conducted any experiments on mammals, let alone humans, we can only hypothesize about possible transferability. The lifespan experiments we have conducted are significant due to the large sample size, a significant advantage of the *Drosophila* experimental system. Transfer to humans certainly requires a series of corresponding human studies under controlled conditions, in which confounding factors, such as excessive salt concentration, have to be ruled out. Despite these reservations, we firmly believe that SEEs have considerable health-promoting potential for humans.

## 5. Conclusions

In conclusion, the aqueous *Salicornia* extract we used in this study shows a vast, sex-specific lifespan extension in *Drosophila*. This opens a broad field of application for use as a human nutritional supplement. On the other hand, mechanistic studies can provide information about the mode of action of the life extension and thus provide a crucial first step toward identifying the life-extending substances of the extract. One strategy for identifying the substances responsible for the life-prolonging effects is to test the most promising candidates at physiological concentrations to determine their potency in increasing the lifespan of *Drosophila*. Priority should be given to substances specifically found in the SEE that have already been linked to health-promoting effects. These include phenolic substances and low-molecular-weight metabolic intermediates, in particular. The molecular basis of the sex-specific effect of life extension can also be elucidated in the future.

## Figures and Tables

**Table 1:** Lifespan extension of different fly strains in response to SEE application.

Fly	Lifespan Extension (%)	*p*-Value
*w*^1118^ male 0.2%	-	0.15
*w*^1118^ female 0.05%	-	0.90
*w*^1118^ female 0.2%	36.1	<0.0001
*yw* female 0.2%	16.1	<0.0001

**Table 2:** Comparison of induced phenotypes and underlying mechanisms of feeding *Salicornia europea* (current study), *Eisenia bicyclis*, and *Sacchoriza polyschides* extracts. − means no effect, + mild effect, ++ strong effect, +++ very strong effect. HF means High Fat diet, HS means High Sugar diet; for the latter two ++ means strong reversion of the negative effects caused by these diets.

Algal Extract	Lifespan-Extension	Sex-Specificity	Tor-Dependent	Foxo-Dependent	Starvation	Body Fat Reduction	HF	HS
*Saliconia*	+++	++	+++	+	−	++	++	−
*Eisenia*	++	++	+++	++	−	−	−	++
*Sacchoriza*	+	−	+++	?	+ −	++		

## Data Availability

The original contributions presented in this study are included in the article/Supplementary Materials. Further inquiries can be directed to the corresponding author.

## Supplementary Materials

The following supporting information can be downloaded at: https://www.mdpi.com/article/10.3390/nu17193065/s1, Table S1: List of lifespan experiments performed in the current manuscript.

## Abbreviations

The following abbreviations are used in this manuscript:

SEE	*Salicornia europaea* extract
rpm	Rounds per minute
TAG	Triacylglycerol
