# Supplementary material for: Sex-Specific Lifespan Extension and Anti-Obesogenic Effects of Salicornia europaea Extract Through Tor Signaling Modulation in Drosophila
Source: Nutrients. 2025 Sep 25;17(19):3065. doi: 10.3390/nu17193065 (PMC12525960; doi:10.3390/nu17193065)
Supplement: Supplementary file 1 [file nutrients-17-03065-s001.zip › nutrients-3837197-supplementary.pdf]

Tahan Zadeh et al.

Table S1: List of lifespan experiments performed in the current manuscript.

Figure	Genotype	Treatment	SEE conc	% lifespan	p	N
1A	w^{1118}	SEE	0.05	-	0.9	102/100
1B	w^{1118}	SEE	0.2	36.7	<0.0001	183/195
1C	w^{1118}	SEE	0.2	-	0.69	101/114
1D	yw	SEE	0.2	18.8	<0.0001	114/118
2E	w^{1118}	Starv/SEE	0.2	-9.8	<0.0001	115101
2F	w^{1118}	dessi/SEE	0.2	-	0.063	111/96
4A	w^{1118}	HSD	0.2	-	0.74	115/112
4B	w^{1118}	HFD	0.2	16.7	0.014	114/114
4D	w^{1118}	Ox. stress	0.2	-	0.23	108/113
5A	Sirt2-KO	SEE	0.2	10.8	<0.0001	110/119
5B	Tor-KO	SEE	0.2	-	0.32	105/111
5C	Foxo-KO	SEE	0.2	14.3	0.0085	114/100
6B	EC-Tor^{DN}	SEE	0.2	14.3	0.062	107/89
6C	EC-S6K-RNAi	SEE	0.2	22.7	<0.0001	114/108
6D	ISC-Tor^{DN}	SEE	0.2	10.7	0.003	57/53
6E	ISC-S6K-RNAi	SEE	0.2	0	0.0094	127/121
7A	FB-Tor^{DN}	SEE	0.2	46.7	<0.0001	68/96
7B	FB-S6K-RNAi	SEE	0.2	67.7	<0.0001	79/71

SEE, *Salicornia europeae* extract, Starv – starvation, dessi – dessication, HSD – High sugar diet, HFD – High fat diet, Ox. Stress -paraquat treatment, EC-Tor^{DN} – NP1-Gal4 X UAS-TorDN, EC-S6K-RNAi - NP1-Gal4 X UAS-S6K-RNAi, ISC-Tor^{DN} – esg-Gal4 X UAS-TorDN, ISC-S6K-RNAi - esg-Gal4 X UAS-S6K-RNAi, FB-Tor^{DN} – inducible fat body driver X UAS-TorDN, FB-S6K-RNAi - inducible fat body driver X UAS-S6K-RNAi.
